# (*E*)-2-[2-(4-Carb­oxy­phen­yl)ethen­yl]-8-hydroxy­quinolin-1-ium chloride ethanol monosolvate

**DOI:** 10.1107/S1600536813030274

**Published:** 2013-11-13

**Authors:** Mathias M. Schulze, Wilhelm Seichter, Edwin Weber

**Affiliations:** a Institut für Organische Chemie, TU Bergakademie Freiberg, Leipziger Strasse 29, D-09596 Freiberg/Sachsen, Germany

## Abstract

In the title compound, C_18_H_14_NO_3_^+^·Cl^−^·CH_3_CH_2_OH, the dihedral angle formed by the mean planes of the quinolinium and benzene rings is 3.4 (1)°, while the carb­oxy substituent is tilted at an angle of 4.8 (1)° with respect to the benzene ring. There is a short N—H⋯O contact in the cation. In the crystal, due to the planar mol­ecular geometry, two-dimensional aggregates are formed parallel to (221) *via* C—H⋯O, C—H⋯Cl, O—H⋯Cl and N—H⋯Cl hydrogen bonds. Inter­layer association is accomplished by O—H_ethanol_⋯Cl and O—H⋯O_ethanol_ hydrogen bonds and π–π stacking inter­actions [centroid–centroid distances vary from 3.6477 (12) to 3.8381 (11) Å]. A supra­molecular three-dimensional architecture results from a stacked arrangement of layers comprising the ionic and hydrogen-bonded components.

## Related literature

For metal-organic framework construction, see: MacGillivray (2010 ▶); Noro & Kitagawa (2010 ▶). For complexation of quinolin-8-ol and its derivatives, see: Albrecht *et al.* (2008 ▶); Weber & Vögtle (1975 ▶). For coordination behavior of carb­oxy­lic groups, see: Kitagawa *et al.* (2004 ▶); Böhle *et al.* (2011 ▶). For the preparative method used for the synthesis of the title compound, see: Yuan *et al.* (2012 ▶). For related structures of quinolinol derivatives, see: Tan (2007 ▶); Zinczuk *et al.* (2008 ▶). For non-classical hydrogen bonds, see: Desiraju & Steiner (1999 ▶). For π–π stacking inter­actions, see: James (2004 ▶).
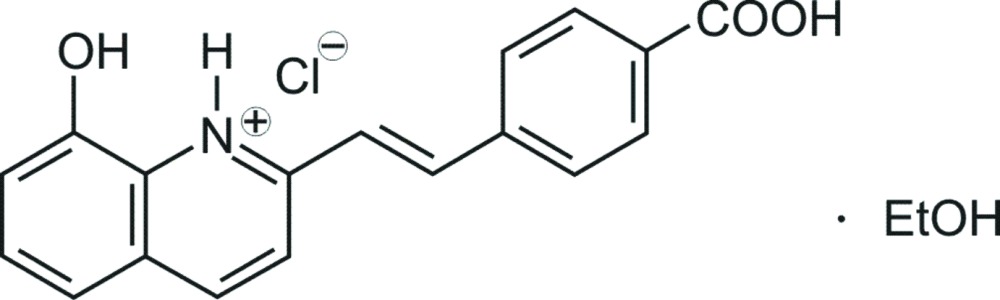


## Experimental

### 

#### Crystal data

C_18_H_14_NO_3_^+^·Cl^−^·C_2_H_6_O
*M* *_r_* = 373.82
Triclinic, 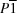

*a* = 9.6841 (2) Å
*b* = 9.7030 (2) Å
*c* = 10.8456 (3) Å
α = 67.516 (1)°
β = 74.957 (1)°
γ = 86.249 (1)°
*V* = 908.72 (4) Å^3^
*Z* = 2
Mo *K*α radiation
μ = 0.24 mm^−1^
*T* = 173 K
0.28 × 0.19 × 0.05 mm

#### Data collection

Bruker APEXII CCD area-detector diffractometer
Absorption correction: multi-scan (*SADABS*; Bruker, 2007 ▶) *T*_min_ = 0.937, *T*_max_ = 0.988
19296 measured reflections
3596 independent reflections
2790 reflections with *I* > 2σ(*I*)
*R*_int_ = 0.022

#### Refinement

*R*[*F* ^2^ > 2σ(*F* ^2^)] = 0.048
*wR*(*F* ^2^) = 0.147
*S* = 1.10
3596 reflections
243 parameters
H atoms treated by a mixture of independent and constrained refinement
Δρ_max_ = 0.43 e Å^−3^
Δρ_min_ = −0.32 e Å^−3^

### 

Data collection: *APEX2* (Bruker, 2007 ▶); cell refinement: *SAINT* (Bruker, 2007 ▶); data reduction: *SAINT*; program(s) used to solve structure: *SHELXS97* (Sheldrick, 2008 ▶); program(s) used to refine structure: *SHELXL97* (Sheldrick, 2008 ▶); molecular graphics: *ORTEP-3 for Windows* (Farrugia, 2012 ▶); software used to prepare material for publication: *SHELXTL* (Sheldrick, 2008 ▶).

## Supplementary Material

Crystal structure: contains datablock(s) I, New_Global_Publ_Block. DOI: 10.1107/S1600536813030274/su2663sup1.cif

Structure factors: contains datablock(s) I. DOI: 10.1107/S1600536813030274/su2663Isup2.hkl

Supplementary material file. DOI: 10.1107/S1600536813030274/su2663Isup3.cml

Additional supplementary materials:  crystallographic information; 3D view; checkCIF report

## Figures and Tables

**Table 1:** Hydrogen-bond geometry (Å, °)

*D*—H⋯*A*	*D*—H	H⋯*A*	*D*⋯*A*	*D*—H⋯*A*
N1—H1*A*⋯O1	0.91 (2)	2.27 (2)	2.678 (2)	107 (2)
O1—H1⋯Cl1^i^	0.82	2.26	3.0780 (16)	173
N1—H1*A*⋯Cl1^ii^	0.91 (2)	2.38 (2)	3.2087 (18)	151 (2)
O1*G*—H1*G*⋯Cl1	0.82	2.26	3.076 (3)	179
O2—H2*A*⋯O1*G* ^iii^	0.82	1.85	2.634 (3)	159
C4—H4⋯O3^iv^	0.93	2.46	3.295 (3)	150
C10—H10⋯Cl1^ii^	0.93	2.69	3.446 (2)	138

Symmetry codes: (i) 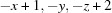; (ii) 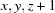; (iii) 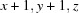; (iv) 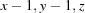.

**Fig. 1.**
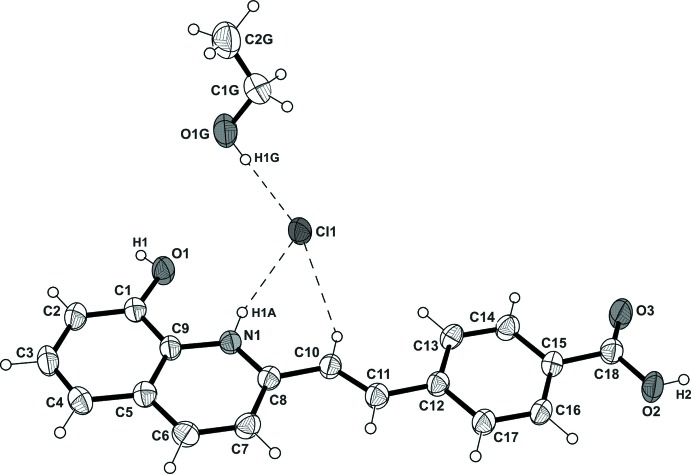
A view of the molecular structure of the title compound, with atom labelling. Displacement ellipsoids are drawn at the 50% probability level. Hydrogen bonds are shown as dashed lines (see Table 1 for details).

**Fig. 2.**
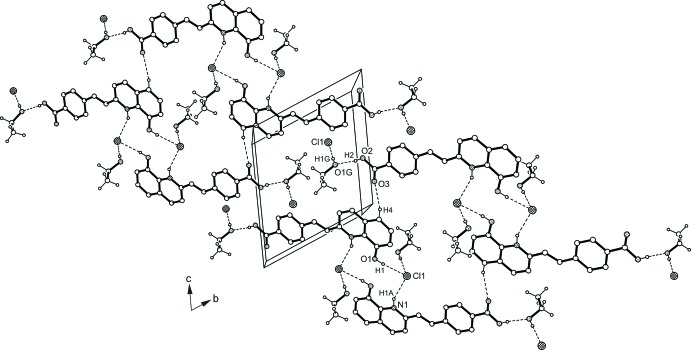
A partial view of the crystal packing of the title compound. Hydrogen bonds are shown as dashed lines within the layer motif (see Table 1 for details).

## supplementary crystallographic information

### 1. Comment

Bifunctional organic ligands have proven very efficient building units in the construction of metal-organic frameworks (MOFs) (MacGillivray, 2010). In particular, one may expect new MOF-architectures from ligands comprising two different coordination sites (Noro *et al.*, 2010). Due to the well known complexation properties of quinolin-8-ol (Albrecht *et al.*, 2008; Weber & Vögtle, 1975) and taking into account the commonly noted coordination behaviour of carboxylic acid groups to various metal ions (Kitagawa *et al.*, 2004; Böhle *et al.*, 2011), corresponding ligands featuring both these structural elements are rated high in this connection. Preparation of a respective hetero bifunctional ligand led to the formation of the title compound. This was isolated as crystals which were found to be a hydrochloride salt containing included ethanol.

In the structure of the title compound (Fig. 1), the principal molecule has an *E* configuration with reference to the ethenyl bond, C10═C11. The overall geometry of this molecule shows approximate planarity with the largest atomic distance from the mean plane of the quinolinium moiety (N1/C1-C9) being -0.018 (1) Å for C8 and 0.011 (2) Å for C9, whereas the phenyl ring (C12-C17 ) is perfectly planar. The dihedral angle between the mean planes of these aromatic building blocks is 3.4 (1) °, while the carboxy substituent (C18/O2/O3) is inclined at an angle of 4.8 (4) ° referring to the phenyl ring. The bond distances within the quinolinium moiety are within expected values (Tan, 2007; Zinczuk *et al.*, 2008).

The chloride ion, Cl1, can be considered as a nodal point within the coordination pattern of the molecules as it is connected with the hydroxy hydrogen [O1—H1···Cl1 2.26 Å, 173 °], the quinolinium hydrogen [N1—H1 A···Cl1 2.38 (2) Å, 151 (2) °] and more weakly (Desiraju & Steiner, 1999) to an ethenyl hydrogen [C10—H10···Cl1 2.69 Å, 138 °] of two different cations. Details are given in Fig. 2 and Table 1. In addition, one molecule of solvent (EtOH) is coordinated by its hydroxy hydrogen to the anion [O1G—H1G···Cl1 2.26 Å, 179 °; see Table 1 and Fig. 2].

In the crystal, there is a layered arrangement of molecules, which apart from the ionic interactions is stabilized by conventional N-H···O and O-H···O hydrogen bonds (Fig. 2 and Table 1). Moreover, in the stacking direction of the molecular layers the mean distance of 3.50 Å between consecutive molecules and the overlap of their aromatic units suggest the presence of π–π interactions (James, 2004). These include Cg1···Cg1^i^ = 3.7477 (12) Å, normal distance = 3.3605 (8) Å, slippage = 1.659 Å; Cg1···Cg2^i^ = 3.8381 (11) Å; Cg1···Cg3^ii^ = 3.6477 (12) Å; Cg2···Cg3^ii^ = 3.7241 (12) Å [Cg1, Cg2 and Cg3 are the centroids of rings C5-C8/N1/C9, C1-C5/C9 and C12-C17, respectively; symmetry codes: (i) -x+1, -y, -z+2; (ii) -x+1, -y+1, -z+2].

### 2. Experimental

The title compound was synthesized *via* Knoevenagel type condensation (Yuan *et al.*, 2012) using 8-hydroxyquinaldine (320 mg, 2.0 mmol) and 4-formylbenzoic acid (1.20 g, 8.0 mmol) in acetic anhydride (100 ml). The mixture was stirred for 30 h under reflux. After removal of the solvent, the residue was dissolved in 100 ml of pyridine/water (*v*/*v* = 4:1) and heated at 373 K for 1 h. Evaporation of the solvent under vacuum and purification of the crude product by recrystallization from ethanol and treatment with hydrochloric acid (37%) yielded 370 mg (63%) of the title compound as brown crystals. The *E* configuration of the compound was confirmed by ^1^H NMR analysis (ethenylene protons); M. p. = 514 K.; MS (ESI) *m/z*: found 292.0 [*M*+H]+; calc. for C_18_H_18_NO_3_ 291.09. Spectroscopic data, including IR and ^1^H and ^13^C NMR, for the title compound are available in the archived CIF.

### 3. Refinement

Crystal data, data collection and structure refinement details are summarized in Table 1. The NH hydrogen was located in a difference Fourier map and freely refined. All other H atoms were positioned geometrically and constrained to ride on their respective parent atoms: O—H = 0.82 Å, C—H = 0.93, 0.96 and 0.97 Å for aryl/ethenyl, methylene and methyl H atoms, respectively, with *U*_iso_(H) = 1.5*U_eq_*(C-methyl and O), and = 1.2*U_eq_*(C) for other H atoms.

### Crystal data

**Table:**

C_18_H_14_NO_3_^+^·Cl^−^·C_2_H_6_O	*Z* = 2
*M**_r_* = 373.82	*F*(000) = 392
Triclinic, *P*1	*D*_x_ = 1.366 Mg m^−^^3^
Hall symbol: -P 1	Mo *K*α radiation, λ = 0.71073 Å
*a* = 9.6841 (2) Å	Cell parameters from 6622 reflections
*b* = 9.7030 (2) Å	θ = 2.3–28.5°
*c* = 10.8456 (3) Å	µ = 0.24 mm^−^^1^
α = 67.516 (1)°	*T* = 173 K
β = 74.957 (1)°	Plate, colourless
γ = 86.249 (1)°	0.28 × 0.19 × 0.05 mm
*V* = 908.72 (4) Å^3^	

### Data collection

**Table:**

Bruker APEXII CCD area-detector diffractometer	3596 independent reflections
Radiation source: fine-focus sealed tube	2790 reflections with *I* > 2σ(*I*)
Graphite monochromator	*R*_int_ = 0.022
phi and ω scans	θ_max_ = 26.1°, θ_min_ = 2.3°
Absorption correction: multi-scan (*SADABS*; Bruker, 2007)	*h* = −11→11
*T*_min_ = 0.937, *T*_max_ = 0.988	*k* = −12→12
19296 measured reflections	*l* = −13→13

### Refinement

**Table:**

Refinement on *F*^2^	Primary atom site location: structure-invariant direct methods
Least-squares matrix: full	Secondary atom site location: difference Fourier map
*R*[*F*^2^ > 2σ(*F*^2^)] = 0.048	Hydrogen site location: inferred from neighbouring sites
*wR*(*F*^2^) = 0.147	H atoms treated by a mixture of independent and constrained refinement
*S* = 1.10	*w* = 1/[σ^2^(*F*_o_^2^) + (0.0792*P*)^2^ + 0.2177*P*] where *P* = (*F*_o_^2^ + 2*F*_c_^2^)/3
3596 reflections	(Δ/σ)_max_ = 0.001
243 parameters	Δρ_max_ = 0.43 e Å^−^^3^
0 restraints	Δρ_min_ = −0.32 e Å^−^^3^

### Special details

**Table:**

Experimental. Spectroscopic data for the title compound:IR (KBr, cm^-1^) 3354, 2810, 2537, 1673, 1284, 1194, 882, 747, 541. ^1^H NMR (500 MHz, DMSO-d_6_) 7.07 (d, ^3^J_HH_ = 7.3 Hz, 1 H), 7.30 (d, ^3^J_HH_ = 8.0 Hz, 1 H), 7.36 (t, ^3^J_HH_ = 7.8 Hz, 1 H), 7.52 (d, ^3^J_HH_ = 16.2 Hz, 1 H), 7.80–7.68 (m, 3H), 7.98 (d, ^3^J_HH_ = 8.0 Hz, 2 H), 8.13–8.07 (m, 1H), 8.21 (d, ^3^J_HH_ = 8.5 Hz, 1 H), 9.39 (br s, 1 H). ^13^C NMR (126 MHz, DMSO-d_6_) 110.9, 117.4, 120.9, 126.9, 127.1, 127.7, 129.2, 129.8, 130.1, 130.4, 133.1, 136.4, 140.6, 152.8, 152.9, 167.0.
Geometry. All e.s.d.'s (except the e.s.d. in the dihedral angle between two l.s. planes) are estimated using the full covariance matrix. The cell e.s.d.'s are taken into account individually in the estimation of e.s.d.'s in distances, angles and torsion angles; correlations between e.s.d.'s in cell parameters are only used when they are defined by crystal symmetry. An approximate (isotropic) treatment of cell e.s.d.'s is used for estimating e.s.d.'s involving l.s. planes.
Refinement. Refinement of *F*^2^ against ALL reflections. The weighted *R*-factor *wR* and goodness of fit *S* are based on *F*^2^, conventional *R*-factors *R* are based on *F*, with *F* set to zero for negative *F*^2^. The threshold expression of *F*^2^ > σ(*F*^2^) is used only for calculating *R*-factors(gt) *etc*. and is not relevant to the choice of reflections for refinement. *R*-factors based on *F*^2^ are statistically about twice as large as those based on *F*, and *R*- factors based on ALL data will be even larger.

### Fractional atomic coordinates and isotropic or equivalent isotropic displacement parameters (Å^2^)

**Table:**

	*x*	*y*	*z*	*U*_iso_*/*U*_eq_	
O1	0.43522 (18)	−0.02294 (17)	1.40451 (15)	0.0570 (4)	
H1	0.4203	−0.0953	1.4778	0.085*	
O2	1.15248 (18)	1.03920 (17)	0.62269 (16)	0.0589 (4)	
H2A	1.2170	1.0978	0.6095	0.088*	
O3	1.1812 (2)	0.9480 (2)	0.83527 (18)	0.0734 (5)	
N1	0.49743 (17)	0.17149 (17)	1.14211 (16)	0.0368 (4)	
H1A	0.534 (2)	0.175 (2)	1.210 (2)	0.040 (5)*	
C1	0.3645 (2)	−0.0445 (2)	1.3210 (2)	0.0424 (5)	
C2	0.2652 (2)	−0.1571 (2)	1.3597 (2)	0.0483 (5)	
H2	0.2427	−0.2267	1.4500	0.058*	
C3	0.1975 (2)	−0.1685 (2)	1.2652 (2)	0.0507 (5)	
H3	0.1303	−0.2460	1.2942	0.061*	
C4	0.2263 (2)	−0.0699 (2)	1.1318 (2)	0.0467 (5)	
H4	0.1795	−0.0797	1.0706	0.056*	
C5	0.3287 (2)	0.0480 (2)	1.0878 (2)	0.0397 (4)	
C6	0.3686 (2)	0.1561 (2)	0.9524 (2)	0.0443 (5)	
H6	0.3251	0.1523	0.8866	0.053*	
C7	0.4694 (2)	0.2659 (2)	0.9156 (2)	0.0436 (5)	
H7	0.4935	0.3365	0.8258	0.052*	
C8	0.5372 (2)	0.2725 (2)	1.0140 (2)	0.0376 (4)	
C9	0.39677 (19)	0.0587 (2)	1.1840 (2)	0.0369 (4)	
C10	0.6483 (2)	0.3809 (2)	0.9871 (2)	0.0393 (4)	
H10	0.6877	0.3711	1.0596	0.047*	
C11	0.6986 (2)	0.4928 (2)	0.8682 (2)	0.0431 (5)	
H11	0.6599	0.5016	0.7956	0.052*	
C12	0.8098 (2)	0.6041 (2)	0.8404 (2)	0.0391 (4)	
C13	0.8669 (2)	0.6107 (2)	0.9430 (2)	0.0425 (5)	
H13	0.8355	0.5419	1.0330	0.051*	
C14	0.9701 (2)	0.7191 (2)	0.9123 (2)	0.0461 (5)	
H14	1.0076	0.7224	0.9819	0.055*	
C15	1.0182 (2)	0.8230 (2)	0.7788 (2)	0.0399 (4)	
C16	0.9616 (2)	0.8162 (2)	0.6766 (2)	0.0477 (5)	
H16	0.9934	0.8847	0.5865	0.057*	
C17	0.8583 (2)	0.7086 (2)	0.7072 (2)	0.0495 (5)	
H17	0.8205	0.7061	0.6375	0.059*	
C18	1.1257 (2)	0.9416 (2)	0.7505 (2)	0.0455 (5)	
Cl1	0.59814 (7)	0.30785 (6)	0.33293 (5)	0.0594 (2)	
O1G	0.3141 (2)	0.2791 (2)	0.5512 (3)	0.1029 (8)	
H1G	0.3894	0.2874	0.4923	0.154*	
C1G	0.2941 (4)	0.4036 (4)	0.5760 (4)	0.1012 (11)	
H1G2	0.3073	0.4879	0.4886	0.121*	
H1G3	0.3670	0.4134	0.6192	0.121*	
C2G	0.1559 (4)	0.4111 (5)	0.6627 (5)	0.1184 (14)	
H1G1	0.0847	0.4259	0.6120	0.178*	
H2G1	0.1567	0.4928	0.6917	0.178*	
H3G1	0.1340	0.3195	0.7422	0.178*	

### Atomic displacement parameters (Å^2^)

**Table:**

	*U*^11^	*U*^22^	*U*^33^	*U*^12^	*U*^13^	*U*^23^
O1	0.0750 (11)	0.0517 (9)	0.0382 (8)	−0.0226 (8)	−0.0202 (8)	−0.0028 (6)
O2	0.0669 (10)	0.0514 (9)	0.0479 (9)	−0.0268 (7)	−0.0149 (8)	−0.0029 (7)
O3	0.0867 (13)	0.0795 (12)	0.0550 (10)	−0.0318 (10)	−0.0257 (9)	−0.0160 (9)
N1	0.0391 (9)	0.0365 (8)	0.0340 (9)	−0.0044 (6)	−0.0107 (7)	−0.0107 (7)
C1	0.0451 (11)	0.0391 (10)	0.0423 (11)	−0.0050 (8)	−0.0097 (9)	−0.0147 (9)
C2	0.0514 (12)	0.0394 (10)	0.0458 (12)	−0.0106 (9)	−0.0069 (10)	−0.0089 (9)
C3	0.0465 (12)	0.0426 (11)	0.0620 (14)	−0.0123 (9)	−0.0104 (10)	−0.0186 (10)
C4	0.0429 (11)	0.0488 (11)	0.0552 (13)	−0.0033 (9)	−0.0171 (10)	−0.0232 (10)
C5	0.0368 (10)	0.0375 (10)	0.0440 (11)	0.0018 (8)	−0.0094 (9)	−0.0152 (8)
C6	0.0444 (11)	0.0506 (11)	0.0417 (11)	0.0014 (9)	−0.0176 (9)	−0.0173 (9)
C7	0.0462 (11)	0.0449 (11)	0.0349 (10)	−0.0007 (9)	−0.0119 (9)	−0.0083 (8)
C8	0.0372 (10)	0.0351 (9)	0.0370 (10)	0.0009 (8)	−0.0068 (8)	−0.0115 (8)
C9	0.0348 (10)	0.0327 (9)	0.0425 (11)	−0.0020 (7)	−0.0072 (8)	−0.0145 (8)
C10	0.0403 (10)	0.0372 (9)	0.0369 (10)	−0.0044 (8)	−0.0094 (8)	−0.0096 (8)
C11	0.0483 (11)	0.0388 (10)	0.0396 (11)	−0.0030 (8)	−0.0125 (9)	−0.0103 (8)
C12	0.0425 (11)	0.0331 (9)	0.0370 (10)	0.0004 (8)	−0.0096 (8)	−0.0085 (8)
C13	0.0463 (11)	0.0387 (10)	0.0331 (10)	−0.0005 (8)	−0.0095 (9)	−0.0034 (8)
C14	0.0488 (12)	0.0470 (11)	0.0377 (11)	−0.0033 (9)	−0.0132 (9)	−0.0086 (9)
C15	0.0387 (10)	0.0348 (10)	0.0401 (11)	−0.0019 (8)	−0.0077 (8)	−0.0086 (8)
C16	0.0594 (13)	0.0412 (10)	0.0331 (10)	−0.0135 (9)	−0.0080 (9)	−0.0039 (8)
C17	0.0622 (13)	0.0466 (11)	0.0363 (11)	−0.0144 (10)	−0.0146 (10)	−0.0079 (9)
C18	0.0451 (11)	0.0444 (11)	0.0439 (12)	−0.0060 (9)	−0.0093 (9)	−0.0133 (9)
Cl1	0.0853 (5)	0.0497 (3)	0.0408 (3)	−0.0215 (3)	−0.0166 (3)	−0.0102 (2)
O1G	0.0707 (13)	0.0620 (12)	0.160 (2)	−0.0253 (10)	0.0134 (13)	−0.0484 (14)
C2G	0.083 (2)	0.107 (3)	0.166 (4)	−0.020 (2)	−0.002 (2)	−0.069 (3)
C1G	0.125 (3)	0.080 (2)	0.099 (3)	−0.030 (2)	−0.002 (2)	−0.0457 (19)

### Geometric parameters (Å, º)

**Table:**

O1—C1	1.349 (2)	C10—C11	1.324 (3)
O1—H1	0.8200	C10—H10	0.9300
O2—C18	1.314 (2)	C11—C12	1.467 (3)
O2—H2A	0.8200	C11—H11	0.9300
O3—C18	1.201 (3)	C12—C17	1.387 (3)
N1—C8	1.330 (2)	C12—C13	1.389 (3)
N1—C9	1.372 (2)	C13—C14	1.383 (3)
N1—H1A	0.91 (2)	C13—H13	0.9300
C1—C2	1.368 (3)	C14—C15	1.387 (3)
C1—C9	1.404 (3)	C14—H14	0.9300
C2—C3	1.391 (3)	C15—C16	1.381 (3)
C2—H2	0.9300	C15—C18	1.491 (3)
C3—C4	1.362 (3)	C16—C17	1.378 (3)
C3—H3	0.9300	C16—H16	0.9300
C4—C5	1.416 (3)	C17—H17	0.9300
C4—H4	0.9300	O1G—C1G	1.327 (4)
C5—C9	1.408 (3)	O1G—H1G	0.8200
C5—C6	1.410 (3)	C2G—C1G	1.438 (5)
C6—C7	1.363 (3)	C2G—H1G1	0.9600
C6—H6	0.9300	C2G—H2G1	0.9600
C7—C8	1.413 (3)	C2G—H3G1	0.9600
C7—H7	0.9300	C1G—H1G2	0.9700
C8—C10	1.449 (3)	C1G—H1G3	0.9700
			
C1—O1—H1	109.5	C10—C11—H11	117.0
C18—O2—H2A	109.5	C12—C11—H11	117.0
C8—N1—C9	124.01 (17)	C17—C12—C13	118.51 (18)
C8—N1—H1A	121.1 (13)	C17—C12—C11	119.03 (18)
C9—N1—H1A	114.8 (13)	C13—C12—C11	122.45 (18)
O1—C1—C2	125.27 (19)	C14—C13—C12	120.38 (18)
O1—C1—C9	116.19 (16)	C14—C13—H13	119.8
C2—C1—C9	118.54 (18)	C12—C13—H13	119.8
C1—C2—C3	120.6 (2)	C13—C14—C15	120.72 (19)
C1—C2—H2	119.7	C13—C14—H14	119.6
C3—C2—H2	119.7	C15—C14—H14	119.6
C4—C3—C2	122.17 (18)	C16—C15—C14	118.88 (18)
C4—C3—H3	118.9	C16—C15—C18	121.90 (18)
C2—C3—H3	118.9	C14—C15—C18	119.19 (18)
C3—C4—C5	118.84 (19)	C17—C16—C15	120.46 (18)
C3—C4—H4	120.6	C17—C16—H16	119.8
C5—C4—H4	120.6	C15—C16—H16	119.8
C9—C5—C6	117.21 (17)	C16—C17—C12	121.04 (19)
C9—C5—C4	118.65 (18)	C16—C17—H17	119.5
C6—C5—C4	124.13 (18)	C12—C17—H17	119.5
C7—C6—C5	121.48 (18)	O3—C18—O2	123.57 (19)
C7—C6—H6	119.3	O3—C18—C15	123.85 (19)
C5—C6—H6	119.3	O2—C18—C15	112.58 (17)
C6—C7—C8	119.97 (18)	C1G—O1G—H1G	109.5
C6—C7—H7	120.0	C1G—C2G—H1G1	109.5
C8—C7—H7	120.0	C1G—C2G—H2G1	109.5
N1—C8—C7	118.15 (17)	H1G1—C2G—H2G1	109.5
N1—C8—C10	116.55 (17)	C1G—C2G—H3G1	109.5
C7—C8—C10	125.30 (18)	H1G1—C2G—H3G1	109.5
N1—C9—C1	119.67 (17)	H2G1—C2G—H3G1	109.5
N1—C9—C5	119.15 (17)	O1G—C1G—C2G	114.6 (3)
C1—C9—C5	121.18 (17)	O1G—C1G—H1G2	108.6
C11—C10—C8	125.54 (19)	C2G—C1G—H1G2	108.6
C11—C10—H10	117.2	O1G—C1G—H1G3	108.6
C8—C10—H10	117.2	C2G—C1G—H1G3	108.6
C10—C11—C12	125.94 (19)	H1G2—C1G—H1G3	107.6
			
O1—C1—C2—C3	179.5 (2)	C6—C5—C9—C1	−179.57 (18)
C9—C1—C2—C3	−0.1 (3)	C4—C5—C9—C1	−0.1 (3)
C1—C2—C3—C4	0.0 (3)	N1—C8—C10—C11	−177.52 (19)
C2—C3—C4—C5	0.1 (3)	C7—C8—C10—C11	2.8 (3)
C3—C4—C5—C9	−0.1 (3)	C8—C10—C11—C12	179.21 (18)
C3—C4—C5—C6	179.41 (19)	C10—C11—C12—C17	175.4 (2)
C9—C5—C6—C7	0.4 (3)	C10—C11—C12—C13	−5.7 (3)
C4—C5—C6—C7	−179.11 (19)	C17—C12—C13—C14	−0.2 (3)
C5—C6—C7—C8	0.5 (3)	C11—C12—C13—C14	−179.04 (18)
C9—N1—C8—C7	1.6 (3)	C12—C13—C14—C15	0.0 (3)
C9—N1—C8—C10	−178.12 (16)	C13—C14—C15—C16	−0.2 (3)
C6—C7—C8—N1	−1.4 (3)	C13—C14—C15—C18	177.77 (18)
C6—C7—C8—C10	178.26 (18)	C14—C15—C16—C17	0.4 (3)
C8—N1—C9—C1	178.55 (17)	C18—C15—C16—C17	−177.4 (2)
C8—N1—C9—C5	−0.8 (3)	C15—C16—C17—C12	−0.6 (3)
O1—C1—C9—N1	1.2 (3)	C13—C12—C17—C16	0.5 (3)
C2—C1—C9—N1	−179.14 (18)	C11—C12—C17—C16	179.4 (2)
O1—C1—C9—C5	−179.47 (17)	C16—C15—C18—O3	−177.2 (2)
C2—C1—C9—C5	0.2 (3)	C14—C15—C18—O3	5.0 (3)
C6—C5—C9—N1	−0.3 (3)	C16—C15—C18—O2	3.2 (3)
C4—C5—C9—N1	179.25 (16)	C14—C15—C18—O2	−174.63 (18)

### Hydrogen-bond geometry (Å, º)

**Table:**

*D*—H···*A*	*D*—H	H···*A*	*D*···*A*	*D*—H···*A*
N1—H1*A*···O1	0.91 (2)	2.27 (2)	2.678 (2)	107 (2)
O1—H1···Cl1^i^	0.82	2.26	3.0780 (16)	173
N1—H1*A*···Cl1^ii^	0.91 (2)	2.38 (2)	3.2087 (18)	151 (2)
O1*G*—H1*G*···Cl1	0.82	2.26	3.076 (3)	179
O2—H2*A*···O1*G*^iii^	0.82	1.85	2.634 (3)	159
C4—H4···O3^iv^	0.93	2.46	3.295 (3)	150
C10—H10···Cl1^ii^	0.93	2.69	3.446 (2)	138

Symmetry codes: (i) −*x*+1, −*y*, −*z*+2; (ii) *x*, *y*, *z*+1; (iii) *x*+1, *y*+1, *z*; (iv) *x*−1, *y*−1, *z*.
